# Diseases of the Circulation

**Published:** 1906-03-31

**Authors:** 


### DISEASES OF THE CIRCULATION.

(*Continued from page* 424.)

**Pericarditis.** — In the treatment of effusion,[16] D. Forsyth points out the relief that is given by turning the patient gradually on to his left side, and even still more forward, so that the distended pericardium may rest on the anterior thoracic wall and the lungs may be relieved from its pressure. We must, however, take care that the abdominal respiration is not interfered with, though some patients will require to sit up continuously in bed. Aspiration must not be left till a late stage, but should be undertaken if the dullness and dyspnœa are increasing and the pulse becomes feeble, and especially if the veins are becoming distended and cyanosis appears. He prefers as the point of puncture a spot near the junction of the seventh costal cartilage with the sternum, whence the needle is passed upwards, backwards, and slightly inwards behind the sternum. The fluid should be withdrawn very slowly. The cyanosis and venous distension will often disappear immediately. Dysphagia appears to be a rare symptom. Saundby [17] has only met with one instance in twenty years. Walshe and Stokes fifty years ago had a controversy as to priority of observation with regard to it. Sibson noted 13 cases, and ascribed it to the pressure of a large effusion on the œsophagus. Stokes, however, points out that it may occur when the effusion is scanty, and refers the pain to nerve irritation; while Broadbent would explain it by direct extension of the inflammation from the posterior wall of the pericardium to the œsophagus. In Saundby's case there was only an ounce of fluid, and the pain was one of the earliest symptoms. He therefore favours Broadbent's suggestion that it only occurs when the posterior wall of the pericardium is inflamed.

**Chronic Endocarditis.** — J. S. Thacher [18] has analysed 900 cases in a New York hospital with the view of clearing up some of the relations between malignant and simple endocarditis. No less than 32 per cent. had pyrexia over 100° without any signs of rheumatism or other complications to account for it, and in many this fever was high and long-continued without apparent cause, yet some of these cases recovered. He points out that the distinction between malignant and simple endocarditis is very uncertain, and that the course of the fever is most variable. In the recognition of the close connection between simple and malignant forms he is in harmony with the views of Payne and Poynton in this country.

**Tachycardia.**—In the distinction of true tachycardia from the functional T. J. Morissey [19] thinks that the following signs are in favour of the former condition: (1) A sudden attack, when the pulse reaches its maximum rate almost immediately. (2) The patient, in some cases, is unconscious of the rapid action. (3) The attack lasts the same time on each occasion. (4) The return to the normal rate is sudden. Permanent rapidity may follow upon a surgical operation, especially excision of the larynx, exophthalmic goitre, influenza; but the causes of the true tachycardia, whether paroxysmal and continued are unknown. Some cases seem to follow after a severe shock, and others occur in persons suffering from myocardial degeneration. In many dilatation takes place during an attack, and in a few this is lasting.

[16] Clin. Jour., Jan. 17. [17] Birm. Med. Rev., Jan. [18] Amer. Jour. Med. Sc., Jan. [19] Med. Record, Dec. 2.

